# Computational insights and study of drugs for dry eye disease through QSPR and MCDM approaches using topological indices

**DOI:** 10.1038/s41598-025-04174-2

**Published:** 2025-07-01

**Authors:** Shanmukha M C, Kirana B, Usha A, Shilpa K C

**Affiliations:** 1 Department of Mathematics, PES Institute of Technology and Management, Shivamogga, 577204 India; 2 Department of Mathematics, KVG College of Engineering, Sullia, 574327 India; 3 Department of Mathematics, Alliance University Alliance College of Engineering and Design, Bangalore, 562106 India; 4 Department of AI and ML, PES Institute of Technology and Management, Shivamogga, 577204 India

**Keywords:** Dry eye disease drugs, Degree-based topological indices, QSPR analysis, Multiple linear regression, TOPSIS, VIKOR, Drug discovery, Chemistry, Materials science, Mathematics and computing

## Abstract

Sjögren’s syndrome is a cause of dry eye disease (DED) which leads to discomfort due to the lack of tear in the eye. This study aims at computing various topological indices for the molecular graphs of dry eye disease drugs. Multiple linear regression is applied to validate the relation between seven physicochemical properties and 11 topological indices. QSPR analysis is carried out for topological indices having correlation greater than 0.82 with properties polar surface area, polarizability, boiling point, enthalpy, molar refraction, molar volume, molecular weight and complexity. Furthermore, the multiple-criteria-decision-making (MCDM) techniques TOPSIS, and VIKOR are used to rank the drugs. For the considered properties of drugs under the study, polarizability has shown significant results with high correlation and least RMSE (r = 0.996 & RMSE = 1.419). The analysis revealed that Tacrolimus and Cyclosporine being ranked number 1 and number 22 respectively as identified by TOPSIS and VIKOR.

## Introduction

Across the globe, considerable amount of people are affected by the dysfunctional tear syndrome which is the most common ophthalmic disease. It is also referred to as dry eye disease or keratoconjunctivitis. This happens when an adequate amount of wetness is not produced by the eye which results in the disturbance between the absorption, tear production and the drainage. When the eye produces considerable amount of tear but there is no good lubrication and is unable to protect against infection resulting in the medical condition called dry eye disease (DED)^1^. It is caused due to the various factors concerning the ocular surface and the tear film with varying symptoms and signs. The symptoms vary from mild itching to severe irritation, fatigue in the eye, burning and inflammation in the eye leading to the high damage of the cornea and subsequently loss of vision. The treatment must be customized to each patient based on the severity of the signs and symptoms associated with pathophysiology.

Different medication techniques are available for treating DED. The diagnosis of DED is found approximately in 5% to 30% of the population and the severity of the same increases with age. Lacrimal functional unit (LFU) constitutes ocular surface, lacrimal glands, eyelids, meibomian glands and the connecting nerves. LFU produces the complex structure of tear film whose three main components are the outermost lipid layer, the intermediate aqueous layer and the innermost mucin layer. This tear film is a blend of the other two layers which helps in functioning of lubrication, protection, removal of any foreign body^2,3^. The impact of female hormones on the meibomian and lacrimal glands as well as the ocular surface, dry eye affects more women than males and becomes more prevalent as people age. Research has indicated that female gender carries a $12\%$ to 22$\%$ prevalent risk of acquiring dry eye. Depending on the diagnostic criteria used, the prevalence of DED varies globally and ranges from about 5$\%$ to 50$\%$ in population-based research. Numerous drugs intended to address the underlying causes of the condition as well as its symptoms are used to treat dry eye disease^4–7^.

There are two types of DED which are aqueous deficient type (lacrimal secretion is reduced) and hyper evaporative type(lacrimal secretion is normal but evaporation is excess). The patients experience the same symptoms for the two types of DED like sandy sensation in eyes, redness, burning, itching, scratching sensation, and watery eyes. They develop photophobia over time and finally loss of vision. The sensitivity to light refers to photophobia. People intolerant to bright sunlight, fluorescent light suffer from photophobia which leads to headaches, squinting and discomfort. There is always an improvement in the medicine available for a particular cause. The emergence of new medicines provide a ray of hope to cure the diseases effectively. Therapeutic agents are used as targeted medicine for specific part or parts of the pathophysiology over the decades. This include immune inhibitors in immunotherapy of cancer, new agents in chemotherapy and many such examples related to specific part or parts^8,9^.

In the US, new studies revealed that 16.4 million people were diagnosed with DED and additional 6% have the probability to experience the symptoms and have not been diagnosed. It has been noticed that, it aggravates with age. In reference to this data, we can forecast the existence of DED to take an upward trend as the population ages. The treatments of DED that are conservative include artificial tears, warm compresses with baby products like shampoo. The treatment of DED is given based on the severity and inflammation of the ocular surface, meibomian gland dysfunction and if any other associated ailments^10^. Few techniques of treatment include artificial tears, Topical corticosteroids, Cyclosporin A, Tacrolimus/Pimecrolimus, Tetracyclines, Omega fatty acids, Vitamin A and Macrolides. To prevent this disease, one has to avoid smoking cigarettes, dry heat air and air conditioning. The current medicines for DED have few limitations as most of these are eye drops or emulsions. As they are administered only (1-5)% of the drug is absorbed by the target tissue and the rest is eliminated through lymphatic flow and conjunctival blood. To overcome this, frequency of the drug administration must be followed^11–14^.

Nonsteroidal anti-inflammatory medications (NSAIDs) such as diclofenac, flurbiprofen, ketorolac, nepafenac, and pranoprofen are mostly used to treat pain and inflammation, especially in ocular applications. To treat dry eye disease, CIS-UCA, Lifitegrast, and Rebamipide work by modifying immune responses and promoting the production of mucin. Corticosteroids such as dexamethasone, fluorometholone, rimexolone, and methylprednisolone are used to control inflammation and immunological responses in a variety of medical disorders. Immunosuppressants such as tacrolimus and cyclosporine are used in the treatment of severe dry eye disease and during organ transplantation. Three broad-spectrum antibiotics-minocycline, tetracycline, and doxycycline have anti-inflammatory qualities. One kind of estrogen utilized in hormone replacement therapy is estradiol^15^. An antimalarial medication called hydroxychloroquine is used to treat autoimmune disorders. Rivoglitazone improves insulin sensitivity, which helps control type 2 diabetes. One chemical building block is piperidine.

The study of the drugs of several ailments is of great importance in chemical graph theory and this branch of science uses graph to model a compound and retrieve a lot of data about the compound. Chemical graph theory is a mathematical framework that is useful in many areas of chemistry, including material science, environmental chemistry, and drug creation, for evaluating and comprehending the structure and behaviour of molecules^16^. In a graph, chemical bonds are represented as edges and atoms as vertices. Mathematical and computational techniques aid in the analysis of molecular structures and the prediction of their attributes. A reliable and adaptable tool for bridging mathematics and chemistry is chemical graph theory. Its significance in both theoretical and practical chemistry is demonstrated by its applications in molecular similarity, chemical database searching, QSPR/QSAR, and property prediction. As this discipline develops further, it should help us understand molecular structures and their characteristics better to make progress in materials science, medication development, and other fields of chemistry^17–20^. There are various tools in chemical graph theory of which TI is one of them.

Topological indices(TIs) can be used to quantify the structure of a molecule. These indices are generated using the molecular structure and have been widely used in chemistry and pharmacy. Quantitative structural property/activity relationship, or QSPR/QSAR analysis is one of the major applications of the indices^21–26^. Depending on the algorithm selected, different information can be found using these indices. There are various types of TI such as degree, distance, eigen value, matching and mixed. Topological indices provide a bridge between the abstract mathematical representation of molecular structures and their real world chemical properties. By leveraging graph theory, chemists can gain insights into molecular behavior, aiding in the development of new drugs, materials, and a deeper understanding of chemical processes^27–29^. MCDM offers a structured approach to evaluate the predictions of molecular properties obtained from QSPR analysis. They facilitate a more informed and efficient drug from the selection process to improve successful outcome in drug development^30,31^. Through QSPR, TIs correlate with pharmacological properties that help structural features such as molecular size, branching patterns or hydrophobic to efficacy.

The research on TIs led to the development of over 3000 indices, reflecting the structural properties of graphs. Tamilarasi et al.^32^, developed four novel indices using multiple linear regression. Recently Suresh et al., used multiple linear regression in QSPR analysis of COVID-19 drugs using TIs. They considered the multigraph of drugs instead of simple graphs^33^. Rasheed et al.^34^, discussed novel indices and thermodynamics properties of eye infection therapeutics through QSAR modeling. Zhou et al., studied thermodynamic features of narcotic drugs using degree based topological indices^35^. Hui et al.^36^, characterized nanotubes by implementing MCDM techniques to perform a comparative analysis between them through optimal ranking. Tahreem and Idrees^37^ considered VIKOR method to highlight the effectiveness of this approach in drug prioritization to offer insights for clinical decision making and drug development. Hayat et al.^38^, analysed QSPR results by employing MCDM methods such as TOPSIS, VIKOR and SAW to rank anticancer drugs to obtain rank of lungs disorder drugs for Boiling points and Enthalpy. Li et al.^39^, visualized the QSPR results through the implementation of VIKOR for anticancer drugs. Idrees et al., worked role of topological indices in predictive modelling (Quadratic) and ranking of drugs for treating eye disorders.

Aims and objectives of this work include the following study

- Molecular graphs of drugs used for treating dry eye disease are examined through the computation of various topological indices (TIs).
- The relationship between 11 topological indices and seven key physicochemical properties is assessed using multiple linear regression.
- Topological indices showing a high correlation coefficient with these properties are used to construct QSPR models.
- To identify the most effective drug among those studied, multi-criteria-decision-making (MCDM) techniques such as TOPSIS and VIKOR are applied for ranking.

## Materials and methods

Chemists and pharmacists utilize the data regarding the physicochemical properties of the compounds using methods that include QSPR, QSTR and QSAR analysis to create novel drugs. These studies offer a systematic approach to understand the features of drugs that improve the ability to target specific aspects of the illness. To design a drug, the targeted qualities and the features of the drugs are considered in which the QSPR analysis and topological indices come handy. The constituent of a drug is chosen based on the availability of data pertaining to that compound and their properties and structural information to compute topological indices.

In this work, we analyse the seven physicochemical properties of 22 drugs for dry eye disease and 11 topological indices using Multiple linear regression. The first Zagreb index, second Zagreb index^40^, Harmonic index^41^, Hyper Zagreb index^42^, Forgotten index^43^, Atom-bond connectivity index^44^, Randic index^45^ , Sum-connectivity index^46^, Geometric-arithmetic index^47^, Sombor index^48^ and Nirmala index^49^ are computed. The above mentioned indices (Table 1) are defined as follows

**Fig. 1:**
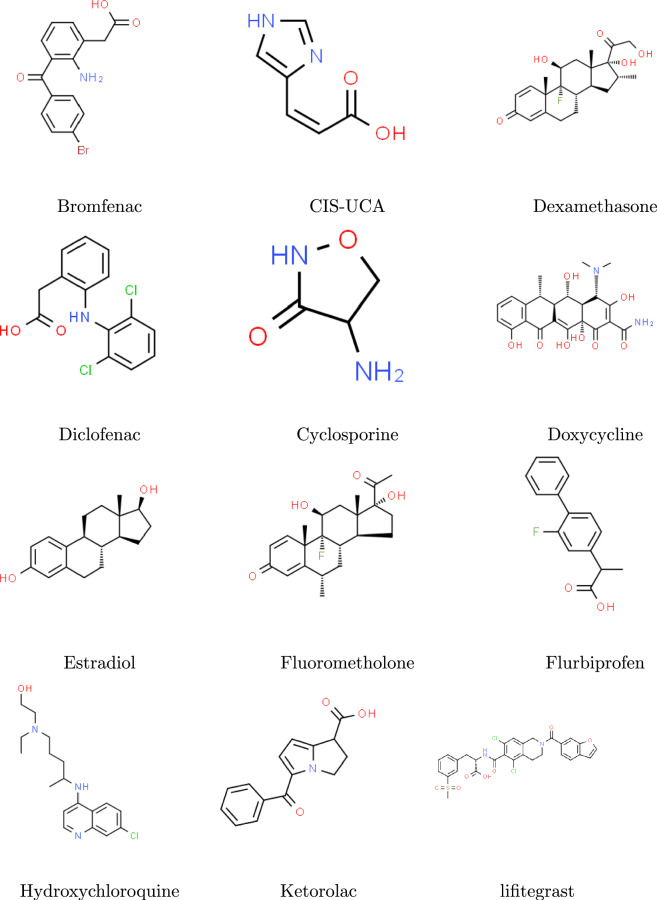

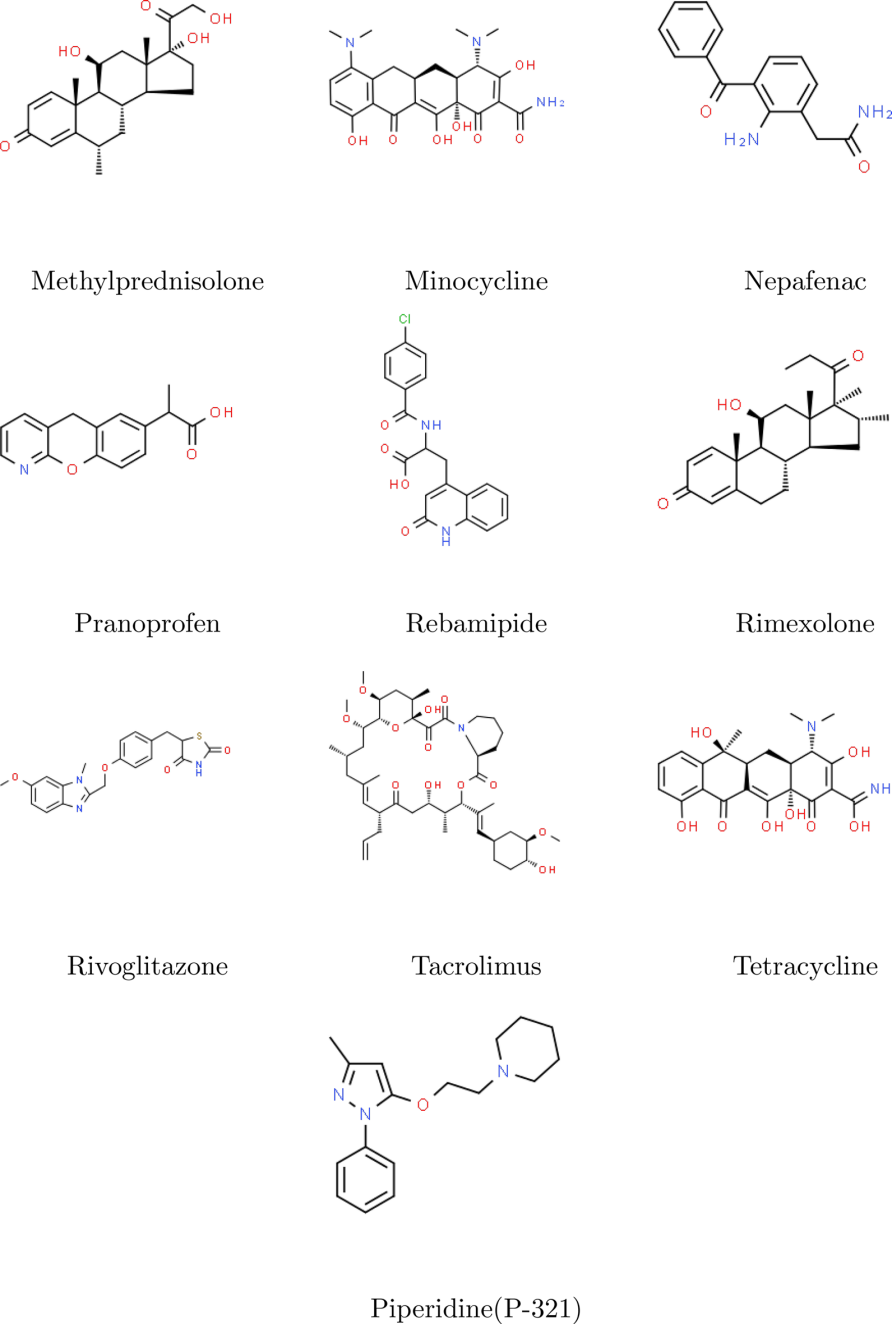
Molecular structures of various drugs for dry eye disease.

**Table 1:** List of various degree-based topological indices.

First and second Zagreb indices		
$$M_{1}(G)=\mathop {\sum }\limits _{u\upsilon \in E(G)}(d_{u}+d_{\upsilon })$$		(1)
$$M_{2}(G)=\mathop {\sum }\limits _{u\upsilon \in E(G)}(d_{u}\times d_{\upsilon })$$		(2)
Harmonic index		
$$H(G)= \mathop {\sum }\limits _{u\upsilon \in E(G)}\frac{2}{d_{u}+d_{\upsilon }}$$		(3)
Hyper Zagreb index		
$$HM(G)=\mathop {\sum }\limits _{u\upsilon \in E(G)}(d_{u}+d_{\upsilon })^2$$		(4)
Forgotten topological index		
$$F(G)=\mathop {\sum }\limits _{u\upsilon \in E(G)}((d_{u})^{2}+(d_{\upsilon })^{2})$$		(5)
Atom-bond connectivity index		
$$ABC(G)=\mathop {\sum }\limits _{u\upsilon \in E(G)}\sqrt{\frac{d_{u}+d_{\upsilon }-2}{d_{u}\times d_{\upsilon }}}$$		(6)
Randi$\acute{c}$ index		
$$R(G)= \mathop {\sum }\limits _{u\upsilon \in E(G)}\frac{1}{\sqrt{d_{u}\times d_{\upsilon }}}$$		(7)
Sum-connectivity index		
$$SC(G)= \mathop {\sum }\limits _{u\upsilon \in E(G)}\frac{1}{\sqrt{d_{u}+d_{\upsilon }}}$$		(8)
Geometric arithmetic index		
$$GA(G)= \mathop {\sum }\limits _{u\upsilon \in E(G)}\frac{2\sqrt{d_{u}\times d_{\upsilon }}}{d_{u}+d_{\upsilon }}$$		(9)
Sombor index		
$$SO(G)= \mathop {\sum }\limits _{u\upsilon \in E(G)}\sqrt{(d_{u}^2+d_{\upsilon }^2)}$$		(10)
Nirmala index		
$$N(G)= \mathop {\sum }\limits _{u\upsilon \in E(G)}\sqrt{(d_{u}+d_{\upsilon })}$$		(11)

A molecular structure of chemical compound is represented by simple graph (molecular graphs) denoted by $G=(V,E)$ where *V* and *E* are the set of vertices and edges respectively. In this work, $d_u$ represents the degree of the vertex *u* belonging to *V*^50–53^. The considered drugs are Bromfenac, CIS-UCA, Dexamethasone, Diclofenac, Cyclosporine, Doxycycline, Estradiol, Fluorometholone, Flurbiprofen, Hydroxychloroquine, Ketorolac, Lifitegrast, Methylprednisolone, Minocycline, Nepafenac, Pranoprofen, Rebamipide, Rimexolone, Rivoglitazone, Tacrolimus, Tetracycline, Piperidine with their properties such as boiling point ($BP ^\circ$ C at 760 mmHg), molar refractivity ($MR \,\ cm^3$), polarizability ($P \,\ 10^{-24}cm^3$), molar volume ($MV \,\ cm^3$), enthalpy of vaporization ($E \,\ kJ/mol$), complexity, molecular weight ($MW \,\ g/mol$) refer to Fig. 1 and Table 2. Physicochemical properties play a significant role in QSPR modeling in the design of drug, metabolism, absorption of the drug, distribution and toxicity prediction. They help in regulating compliance and predicting interactions of the drug in formulating the design which affects the stability and bioavailability of the drug.

### Multiple linear regression

The outcome of a dependent variable is predicted when two are more independent variables are involved using a statistical tool called multiple linear regression. The general equation of multiple linear regression is

$$\begin{aligned}&Y=b_1*I_1+b_2*I_2+b_3*I_3+.....+c \end{aligned}$$

Where, *Y* refers to dependent variable, $b_i$ denotes the coefficients of independent variables *I* and *c* represents regression constant.

There are several types of multiple linear regression models such as enter method (standard method), stepwise method, forward elimination and backward elimination. In this article, enter method of multiple linear regression is used. The properties are taken as dependent variables and topological indices are taken as independent variables. In the multiple regression model *n*: Number of drugs used, *r*: Correlation coefficient, $R^2$: Coefficient of determination, *SE*: Standard error, *F*: Fisher’s statistics, *RMSE*: Root mean square error and *p*: Significant probability.

## Results and discussions

### **Theorem 3.1**

*Let* *G* *be a molecular graph of Diclofenac, then*

$$M_1=94$$, $$M_2=107$$, $$H=8.7$$, $$HM=452$$, $$F=238$$, $$ABC=14.458$$, $$R=9.075$$, $$S=9.302$$, $$GA=19.302$$, $$SO=68.363$$, $$N=43.237$$.

**Fig. 2:**
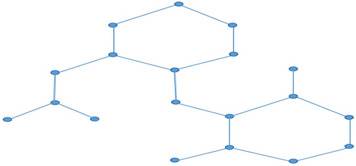
Molecular graph of Diclofenac.

### ***Proof***

From Fig. 2, $\vert V(G)\vert =19$ and $\vert E(G)\vert =20$. They are as follows,

$$\begin{aligned}&E_{1,3}=\left\{ e=u\upsilon \in E(G)\vert d_{u}=1,\,\ d_{\upsilon }=3\right\} ,\,\,\\&E_{2,2}=\left\{ e=u\upsilon \in E(G)\vert d_{u}=2,\,\ d_{\upsilon }=2\right\} , \\&E_{2,3}=\left\{ e=u\upsilon \in E(G)\vert d_{u}=2,\,\ d_{\upsilon }=3\right\} , \,\,\\&E_{3,3}=\left\{ e=u\upsilon \in E(G)\vert d_{u}=3,\,\ d_{\upsilon }=3\right\} , \end{aligned}$$

such that $$\vert E_{1,3} \vert =4,\,\ \vert E_{2,2} \vert =5,\,\ \vert E_{2,3} \vert =8,\,\ \vert E_{3,3} \vert =3$$.

The results are obtained using equations (1)-(11). $\square$

Similarly, the results of other drugs are depicted in Table 3.

**Table 2:** Physicochemical properties for DED drugs.

Drug	BP	MR	P	MV	E	C	MW
Bromfenac	472.23	79.1	31.4	213.5	88.9	366	334.16
CIS-UCA	371.97	36.2	14.4	96.6	75.5	156	138.12
Dexamethasone	508.92	100.2	39.7	296	98	805	392.5
Diclofenac	423.77	76.5	30.3	206.8	70.1	304	296.1
Cyclosporine	281.12	22.5	8.9	79.9	-	92.9	102.09
Doxycycline	753.5	109	43.2	271.1	116.5	956	444.4
Estradiol	395.47	79.5	31.5	232.6	74.2	382	272.4
Fluorometholone	465.47	98.7	39.1	298.9	92.2	787	376.5
Flurbiprofen	380.71	66.6	26.4	203.6	65.8	286	244.26
Hydroxychloroquine	455.39	99	39.2	285.4	83	331	335.9
Ketorolac	414.83	70.5	28	191.2	80.1	376	255.27
Lifitegrast	–	153.7	60.9	416	123.7	1100	615.5
Methylprednisolone	513.91	100.1	39.7	291.5	98.5	754	374.5
Minocycline	743.4	116	46	294.6	122.5	–	–
Nepafenac	470.54	86	29.1	203.4	84.6	337	254.28
Pranoprofen	405.34	69.2	-	195	76.6	346	255.27
Rebamipide	641.76	95.1	37.7	265.8	106.9	598	370.8
Rimexolone	459.38	106.3	42.1	329.2	89.5	749	370.5
Rivoglitazone	729.27	106.5	42.2	280.9	98.4	584	397.4
Tacrolimus	871.7	214.1	84.9	673.1	143.9	1480	804
Tetracycline	745.32	106.9	42.4	266.3	113	971	444.4
Piperidine	400.8	85.4	33.9	252.7	69.1	300	285.4

**Table 3:** Computed TIs for DED drugs.

Drug Name/index	$M_1$	$M_2$	*H*	*HM*	*F*	*ABC*	*R*	*SC*	*GA*	*SO*	*N*
Bromfenac	100	115	9.033	488	258	15.234	9.486	9.71	20.168	72.94	45.686
CIS-UCA	44	46	4.6	196	104	7.289	4.787	4.788	9.651	32.06	20.944
Dexamethasone	168	221	12.747	960	518	22.393	12.963	13.59	29.249	123.985	71.68
Diclofenac	94	107	8.7	452	238	14.458	9.075	9.302	19.302	68.363	43.237
Cyclosporine	32	35	3.133	150	80	5.128	3.304	3.302	6.691	23.435	14.921
Doxycycline	184	237	13.89	1006	532	25.279	14.866	15.507	33.255	134.658	79.837
Estradiol	118	148	9.238	624	328	16.356	9.593	10.269	22.272	85.654	51.899
Fluorometholone	156	201	11.226	874	472	21.428	11.543	12.695	27.376	115.12	66.918
Flurbiprofen	90	104	8.233	438	230	13.71	8.592	8.816	18.342	65.395	41.214
Hydroxychloroquine	110	124	10.8	518	270	17.108	11.134	11.337	23.375	79.519	51.195
Ketorolac	102	123	8.9	402	264	14.934	9.181	9.632	20.476	73.546	46.113
Lifitegrast	226	273	18	1109	587	31.663	18.79	19.854	42.419	159.494	97.947
Methylprednisolone	160	204	11.792	892	480	21.624	12.581	8.85	28.432	117.799	68.886
Minocycline	188	239	14.323	1018	540	26.067	15.333	15.979	34.174	137.766	81.882
Nepafenac	94	108	8.733	454	238	14.417	9.092	8.782	19.342	68.223	43.214
Pranoprofen	102	121	8.833	506	264	15.015	9.147	9.605	20.396	73.826	46.158
Rebamipide	134	155	11.933	654	344	20.184	12.435	12.893	27.047	97.401	61.102
Rimexolone	160	207	11.811	846	480	21.603	12.591	13.228	28.93	117.69	68.869
Rivoglitazone	150	177	13.1	740	386	22.086	13.529	14.207	30.157	108.512	68.015
Tacrolimus	282	325	24.838	1427	750	41.647	26.074	26.697	45.219	205.545	127.279
Tetracycline	178	226	13.471	964	512	24.614	14.406	15.062	32.292	130.425	77.45
Piperidine	106	121	10.166	498	256	16.291	10.326	10.788	22.663	75.987	49.259

**Table 4:** The correlation coefficient *r* between TIs and properties of DED drugs.

	$M_1$	$M_2$	*H*	*HM*	*F*	*ABC*	*R*	*SC*	*GA*	*SO*	*N*
BP	0.853	0.828	0.858	0.827	0.809	0.867	0.834	0.86	**0.876**	0.85	0.862
MR	0.953	0.912	**0.99**	0.91	0.902	0.977	0.893	0.969	0.946	0.949	0.968
P	0.958	0.917	**0.993**	0.915	0.907	0.982	0.894	0.973	0.95	0.954	0.973
MV	0.916	0.87	**0.962**	0.874	0.867	0.943	0.855	0.936	0.889	0.913	0.932
E	0.908	0.891	0.89	0.891	0.883	**0.911**	0.891	0.875	0.901	0.908	0.91
C	0.979	0.98	0.931	0.9805	**0.9814**	0.963	0.902	0.915	0.944	0.9808	0.971
MW	0.97	0.935	**0.99**	0.934	0.924	0.988	0.916	0.976	0.961	0.966	0.98

Table 4 displays the correlation coefficient *R* for TIs against properties of DED drugs.

Table 5 displays the predicted values for the properties of DED drugs.

**Table 5:** Predicted values for properties of DED drugs.

Drug	BP	MR	P	MV	E	C	MW
Bromfenac	458.857	78.630	30.728	221.581	83.021	387.944	293.877
CIS-UCA	273.089	39.986	15.441	123.535	59.369	88.655	146.125
Dexamethasone	496.345	104.903	41.446	315.533	95.335	796.940	394.091
Diclofenac	449.288	75.319	29.371	210.235	80.801	341.385	278.808
Cyclosporine	228.858	24.979	9.743	82.800	–	47.487	95.300
Doxycycline	704.468	109.816	43.040	267.148	115.549	938.447	438.223
Estradiol	527.846	72.056	29.289	202.802	79.213	462.169	271.892
Fluorometholone	511.098	96.131	38.017	276.292	100.934	854.167	390.283
Flurbiprofen	439.629	69.541	27.020	188.198	79.363	328.649	259.511
Hydroxychloroquine	522.831	92.897	35.868	247.125	89.203	360.771	331.784
Ketorolac	398.019	73.397	28.738	199.506	79.488	380.022	254.984
Lifitegrast	–	153.141	60.722	413.868	123.984	1101.611	615.603
Methylprednisolone	516.872	101.122	39.628	291.208	98.834	753.604	371.964
Minocycline	706.548	116.747	46.069	301.952	115.546	–	–
Nepafenac	455.579	74.137	28.429	191.480	83.839	347.325	274.264
Pranoprofen	500.720	71.859	–	194.351	79.836	369.867	268.006
Rebamipide	591.027	103.883	40.950	289.044	95.722	531.537	384.118
Rimexolone	502.036	99.806	40.421	310.120	91.066	740.852	371.263
Rivoglitazone	675.424	109.789	43.685	298.016	98.225	568.658	401.484
Tacrolimus	879.850	213.494	84.718	670.553	144.440	1483.093	804.678
Tetracycline	671.393	110.129	43.797	295.427	108.671	882.825	433.377
Piperidine	395.023	85.339	33.877	253.326	68.560	294.895	284.116

### Regression models

**Model 1**

$$\begin{aligned} BP = & 130.938 + 7.083(M_{2} ) - 58.424(H) + 1.092(HM) - 5.986(F) + 15.083(R) \\ & + 2.502(SC) + 8.844(GA) + 15.475(N).\\                               & n = 21,r = 0.932,R^{2} = 0.869,SE = 74.919,F = 9.964,RMSE = 63.633,p = 0.00 \end{aligned}$$

**Model 2**

$$\begin{aligned} MR = & - 5.883 + 1.652(M_{1} ) - 1.391(M_{2} ) + 8.146(H) - 0.036(HM) + 0.382(F) \\ & + 2.829(ABC) - 0.159(R) + 0.037(SC) + 1.816(GA) - 3.368(N). \\ & n = 22,r = 0.993,R^{2} = 0.985,SE = 6.408,F = 73.25,RMSE = 4.531,p = 0.000. \\ \end{aligned}$$

**Model 3**

$$\begin{aligned} P = & - 2.012 + 0.991(M_{1} ) - 0.857(M_{2} ) + 2.114(H) - 0.015(HM + 0.186(F) - 2.21(ABC) - 0.134(R) \\ & + 0.494(SC) + 0.88(GA) - 0.336(N).\\ & n = 21,r = 0.996, R^{2} = 0.991,SE = 2.057,F = 111.297,RMSE = 1.419,p = 0.000. \\ \end{aligned}$$

**Model 4**

$$\begin{aligned} MV = & 0.829 + 20.337(M_{1} ) - 16.462(M_{2} ) + 23.664(H) - 0.284(HM) + 3.643(F) - 76.508(ABC) \\ & - 4.307(R) + 11.289(SC) + 7.629(GA) + 0.173(N). \\ & n = 22,r = 0.988,R^{2} = 0.977,SE = 24.554,F = 46.274,RMSE = 17.362,p = 0.000. \\ \end{aligned}$$

**Model 5**

$$\begin{aligned} E = & 34.948 - 3.766(M_{1} ) + 2.66(M_{2} ) - 1.387(H) + 0.03(HM) - 0.39(F) \\ & + 32.311(ABC) + 1.77(R) - 3.034(SC) - 1.006(GA) - 5.294(N). \\ & n = 21,r = 0.944,R^{2} = 0.892,SE = 9.735,F = 8.23,RMSE = 6.718,p = 0.001. \\ \end{aligned}$$

**Model 6**

$$\begin{aligned} C = & - 115.791 - 1.752(M_{1} ) + 14.189(M_{2} ) - 55.935(H) - 0.063(HM) - 1.712(F) \\ & + 263.177(ABC) + 3.817(R) - 8.104(SC) - 34.905(GA) - 70.874(N). \\ & n = 21,r = 0.993,R^{2} = 0.986,SE = 59.764,F = 68.631,RMSE = 41.241,p = 0.001. \\ \end{aligned}$$

**Model 7**

$$\begin{aligned} MW = & - 17.309 + 8.481(M_{1} ) - 2.412(M_{2} ) + 12.147(H) + 0.056(HM) + 0.32(F) \\ & + 60.661(ABC) + 1.641(R) + 1.952(SC) + 2.1(GA) - 32.396(N). \\ & n = 21,r = 0.996,R^{2} = 0.993,SE = 18.435,F = 133.996,RMSE = 12.721,p = 0.000. \\ \end{aligned}$$

**Fig. 3:**
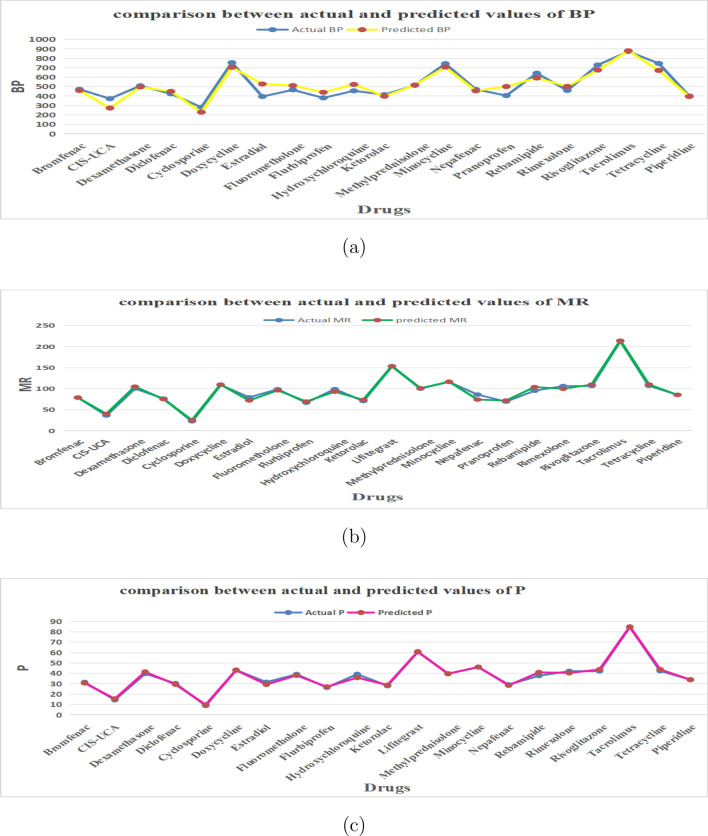

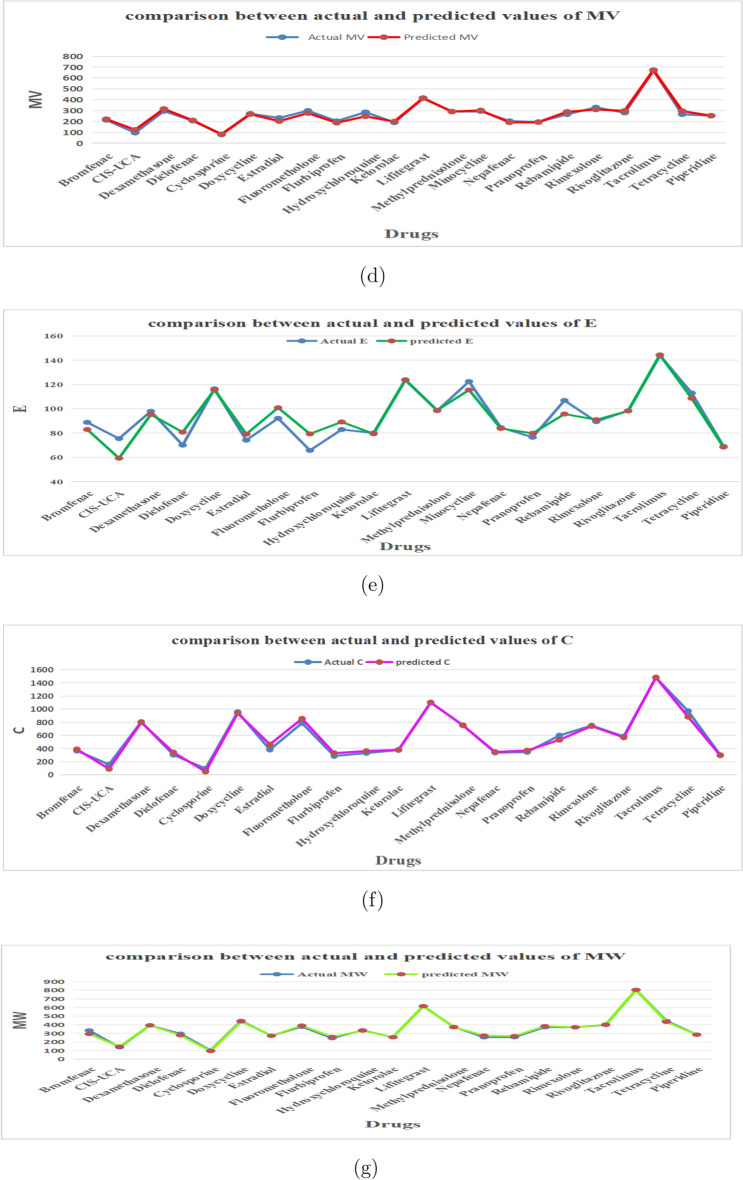
Plots for predicted and actual values of properties of DED drugs.

From the above models, it is observe that each model shows high correlation coefficient, high coefficient of determination, minimal *RMSE* while other parameters are discussed model wise. It is obvious from the minimal *RMSE*, polarizability is found to be the best predictive property among the considered seven properties. Followed by *P* ($RMSE=1.149$), *MR* ($RMSE=4.531$), *E* ($RMSE=6.718$), *MW* ($RMSE=12.721$), *MV* ($RMSE=17.362$) , *C* ($RMSE=41.241$) and *BP* ($RMSE=63.633$) with TIs. For better understanding Tables 2, 5 and Fig. 3 displays the comparison of actual and predicted values for considered drug properties.

### MCDM techniques

**Fig. 4:**
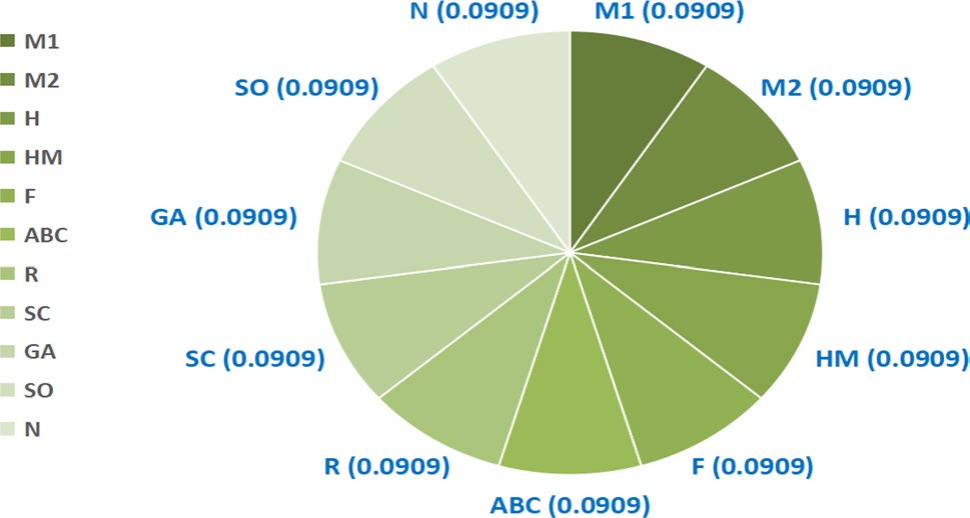
TOPSIS method: allocation of weights.

**Fig. 5:**
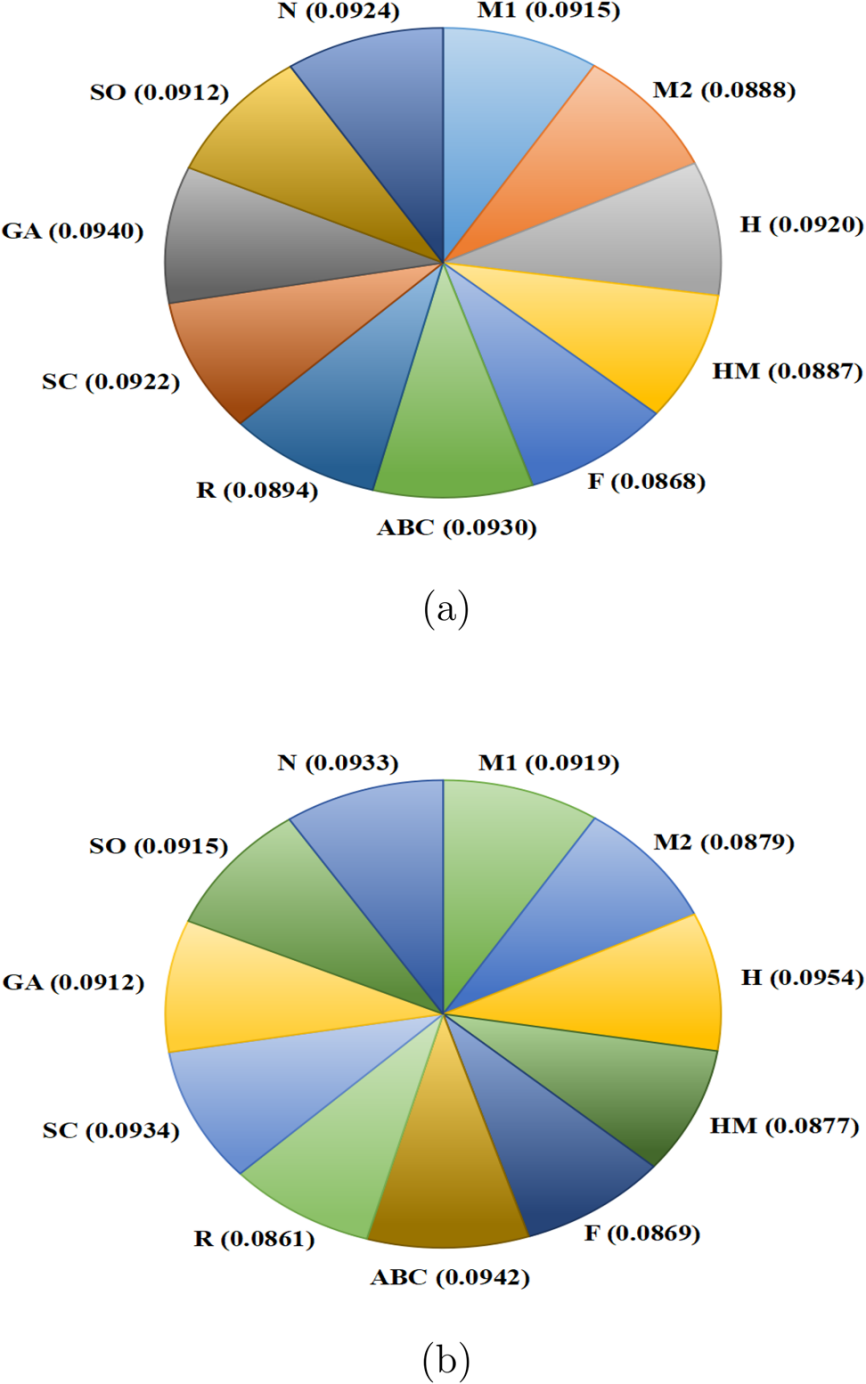
VIKOR method: allocation of weights for BP and MR.

QSPR analysis along with VIKOR and TOPSIS methods provide a relevant approach for evaluating and ranking the compounds based on biological and pharmacological criteria. Multi-Criterion Decision Making techniques TOPSIS and VIKOR are implemented to rank 22 drugs for dry eye disease. In TOPSIS method, weightage is assigned equally (Fig. 4). In VIKOR method, weightage is assigned using ratio method (Fig. 5)^36,37^ and similar allocation of weights for other properties are also studied.

**Table 6:** Computed value $D_i^+,D_i^-, C_i$ and TOPSIS rank of DED drugs.

Drug Name/index	$D_{i}^+$	$D_{i}^-$	$C_{i}$	TOPSIS Rank
Bromfenac	0.0792425	0.0313551	0.2835059	16
CIS-UCA	0.1036562	0.0073225	0.0659810	21
Dexamethasone	0.0523261	0.0596004	0.5324958	6
Diclofenac	0.0817147	0.0289022	0.2612817	18
Cyclosporine	0.1095335	0.0028185	0.0250865	22
Doxycycline	0.0452132	0.0663877	0.5948673	4
Estradiol	0.0786951	0.0349044	0.3072586	14
Fluorometholone	0.0580268	0.0537571	0.4809021	9
Flurbiprofen	0.0836265	0.0269788	0.2439195	20
Hydroxychloroquine	0.0737575	0.0369966	0.3340424	12
Ketorolac	0.0797488	0.0310779	0.2804191	17
Lifitegrast	0.0283203	0.0831992	0.7460505	2
Methylprednisolone	0.0584923	0.0543814	0.4817896	8
Minocycline	0.0433249	0.0681880	0.6114807	3
Nepafenac	0.0819084	0.0287272	0.2596557	19
Pranoprofen	0.0790103	0.0316358	0.2859189	15
Rebamipide	0.0643290	0.0463431	0.4187424	11
Rimexolone	0.0559618	0.0555727	0.4982556	7
Rivoglitazone	0.0576850	0.0530631	0.4791336	10
Tacrolimus	0.0000000	0.1104295	1.0000000	1
Tetracycline	0.0477442	0.0636884	0.5715418	5
Piperidine	0.0760671	0.0346856	0.3131805	13

**Table 7:** Computed values $S_j, R_j, Q_j$ and VIKOR rank of BP.

Drug Name	$S_j$	$R_j$	$Q_j$	VIKOR RANK
Bromfenac	0.715677	0.067254	0.718312	14
CIS-UCA	0.939291	0.087494	0.938633	21
Dexamethasone	0.461794	0.051675	0.507553	6
Diclofenac	0.738272	0.069229	0.740205	16
Cyclosporine	0.992911	0.093951	1.000000	22
Doxycycline	0.397435	0.046415	0.447154	4
Estradiol	0.694148	0.091484	0.836424	20
Fluorometholone	0.514835	0.057709	0.566381	9
Flurbiprofen	0.755738	0.071134	0.759137	19
Hydroxychloroquine	0.665114	0.063136	0.670935	12
Ketorolac	0.719176	0.071193	0.741038	17
Lifitegrast	0.242043	0.028990	0.276170	2
Methylprednisolone	0.512927	0.070362	0.632756	11
Minocycline	0.380585	0.044579	0.428899	3
Nepafenac	0.739766	0.070630	0.748411	18
Pranoprofen	0.712901	0.067855	0.720113	15
Rebamipide	0.578548	0.054712	0.582512	10
Rimexolone	0.496739	0.055229	0.544068	8
Rivoglitazone	0.516895	0.049807	0.525361	7
Tacrolimus	0.000000	0.000000	0.000000	1
Tetracycline	0.421798	0.048192	0.468876	5
Piperidine	0.685785	0.064856	0.690496	13

**Table 8:** Computed values $S_j, R_j, Q_j$ and VIKOR rank of MR.

Drug name	$S_j$	$R_j$	$Q_j$	VIKOR RANK
Bromfenac	0.715852	0.069524	0.724580	14
CIS-UCA	0.939273	0.089024	0.939211	21
Dexamethasone	0.462232	0.053186	0.511304	6
Diclofenac	0.738421	0.070989	0.743616	17
Cyclosporine	0.992878	0.095477	1.000000	22
Doxycycline	0.398018	0.048159	0.452636	4
Estradiol	0.694624	0.091909	0.831116	20
Fluorometholone	0.515224	0.059877	0.573028	9
Flurbiprofen	0.755886	0.073043	0.763170	19
Hydroxychloroquine	0.665297	0.063337	0.666721	12
Ketorolac	0.719314	0.070443	0.731137	16
Lifitegrast	0.242650	0.030079	0.279717	2
Methylprednisolone	0.513637	0.071290	0.631998	11
Minocycline	0.381164	0.046254	0.434175	3
Nepafenac	0.739948	0.071562	0.747387	18
Pranoprofen	0.713107	0.070403	0.727805	15
Rebamipide	0.578827	0.056767	0.588771	10
Rimexolone	0.497231	0.057304	0.550491	8
Rivoglitazone	0.517245	0.051634	0.530876	7
Tacrolimus	0.000000	0.000000	0.000000	1
Tetracycline	0.422339	0.050002	0.474536	5
Piperidine	0.685967	0.065421	0.688047	13

**Fig. 6:**
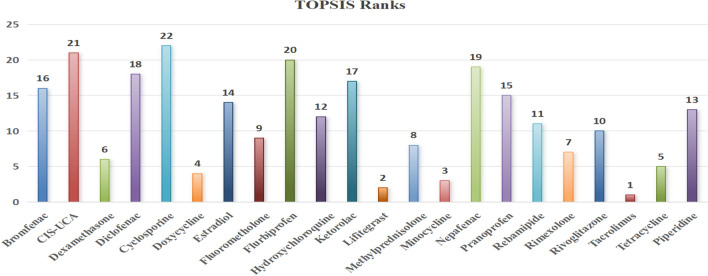
Comparison of TOPSIS ranks.

**Fig. 7:**
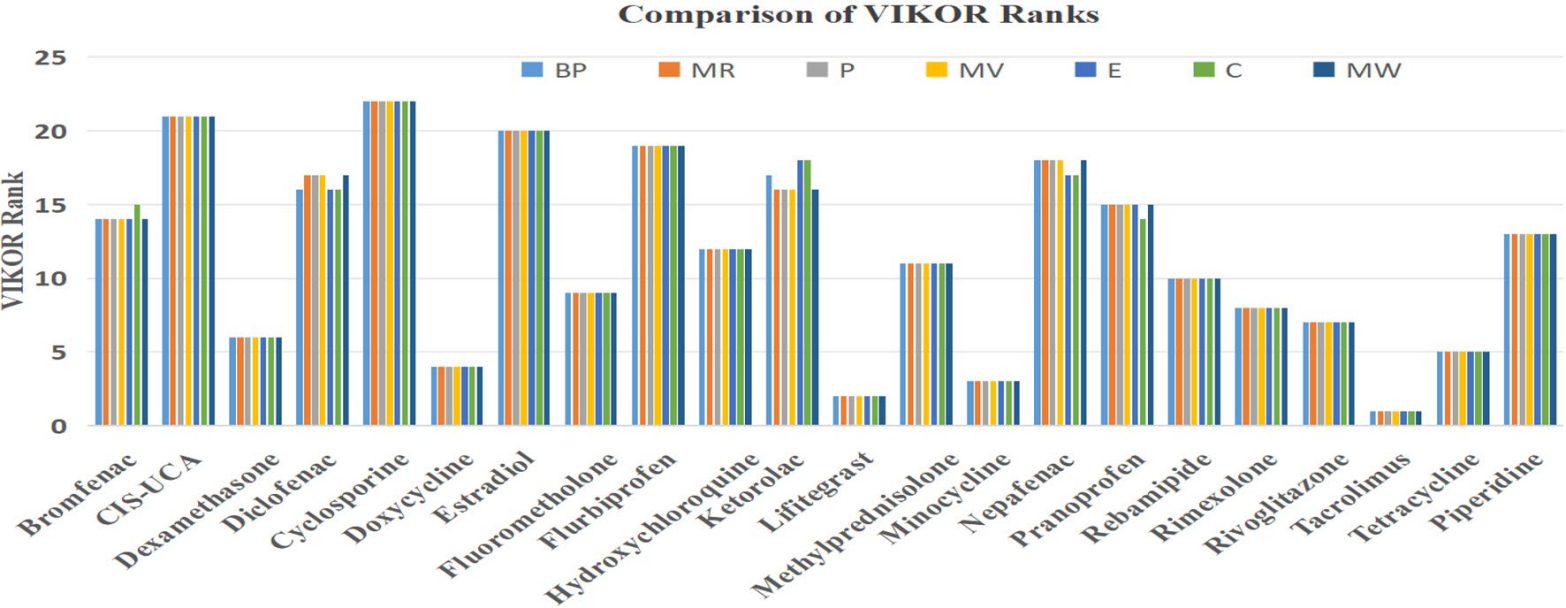
Comparison of VIKOR ranks.

Based on the analysis, considered 22 drugs are ranked using TOPSIS method are tabulated in Table 6 while Tables 7 and 8 display the ranks provided by VIKOR with respect to properties BP and MR. Table 9 gives a detailed comparison of ranks provided by VIKOR for all the drugs with respect to the properties considered in the study. Figures 6 and  7 gives a clear picture of the comparison of the ranks for the considered drugs provided by TOPSIS and VIKOR respectively.

**Table 9:** Comparison of VIKOR rank of all properties.

Drugs	BP	MR	P	MV	E	C	MW
Bromfenac	14	14	14	14	14	15	14
CIS-UCA	21	21	21	21	21	21	21
Dexamethasone	6	6	6	6	6	6	6
Diclofenac	16	17	17	17	16	16	17
Cyclosporine	22	22	22	22	22	22	22
Doxycycline	4	4	4	4	4	4	4
Estradiol	20	20	20	20	20	20	20
Fluorometholone	9	9	9	9	9	9	9
Flurbiprofen	19	19	19	19	19	19	19
Hydroxychloroquine	12	12	12	12	12	12	12
Ketorolac	17	16	16	16	18	18	16
Lifitegrast	2	2	2	2	2	2	2
Methylprednisolone	11	11	11	11	11	11	11
Minocycline	3	3	3	3	3	3	3
Nepafenac	18	18	18	18	17	17	18
Pranoprofen	15	15	15	15	15	14	15
Rebamipide	10	10	10	10	10	10	10
Rimexolone	8	8	8	8	8	8	8
Rivoglitazone	7	7	7	7	7	7	7
Tacrolimus	1	1	1	1	1	1	1
Tetracycline	5	5	5	5	5	5	5
Piperidine	13	13	13	13	13	13	13

## Comparison

It is observed that many of the articles published on the eye disease drugs. The existing work^54,55^ uses linear and quadratic regression models to study QSPR and MCDM techniques such as TOPSIS and SAW methods with limited number of different eye disease drugs and the first ranking is obtained by Acedylcysteine. In this article multiple linear regression is used to study QSPR and the MCDM technique such as TOPSIS and VIKOR methods to rank the considered 22 drugs and the first ranking is obtained by Tacrolimus. The study gives better accuracy compared to existing results in terms of various statistical parameters.

## Conclusion

This study focuses on using multiple linear regression to find the predictive property using various TIs for dry eye disease drugs. By analyzing a dataset of known compounds, MLR helps identify key descriptors that significantly influence the drug’s efficacy. Seven multiple regression models are performed and found to have good correlation, coefficient of determination and a comparatively minimum RMSE. Among these, polarizability shows a correlation of $r=0.996$, $R^2=0.991$ and $RMSE=1.419$. It is stated that polarizability is a good predictive property for the considered TIs. The study is extended using MCDM techniques (VIKOR and TOPSIS) to evaluate and rank 22 DED drugs. The analysis revealed that, Tacrolimus and Cyclosporine being ranked number 1 and number 22 respectively as identified by TOPSIS and VIKOR. Tacrolimus being ranked number one among the considered drugs is an immunosuppressant that reduces inflammation by calming down the over-active immune cells that attack the eye tissues which is similar to steroid functioning. Especially in refractory cases like Sjögren’s syndrome, Tacrolimus is very effective. The analysis demonstrates TOPSIS and VIKOR can effectively guide decision making and further study in the pharmaceutical industry.

## Data Availability

The datasets used and/or analysed during the current study are available from the corresponding author on reasonable request.

## References

[1] Lemp, M. A. & Foulks, G. N. The definition and classification of dry eye disease. *Ocul. Surf.* **5**(2), 75–92 (2007). 17508116 10.1016/s1542-0124(12)70081-2

[2] Gayton, J. L. Etiology, prevalence, and treatment of dry eye disease. *Clin. Ophthalmol* **201**, 405–412 (2009). 10.2147/opth.s5555 PMC2720680 19688028

[3] Messmer, E. M. The pathophysiology, diagnosis, and treatment of dry eye disease. *Deutsches Ärzteblatt Int.* **112**(5), 71 (2015). 10.3238/arztebl.2015.0071 PMC4335585 25686388

[4] Mohamed, H. B., Abd El-Hamid, B. N., Fathalla, D. & Fouad, E. A. Current trends in pharmaceutical treatment of dry eye disease: a review. *Europ. J. Pharmaceut. Sci.* **175**, 106206 (2022). 10.1016/j.ejps.2022.106206 35568107

[5] Kam, K. W. et al. A review on drug-induced dry eye disease. *Indian J. Ophthalmol.* **71**(4), 1263–1269 (2023). 37026257 10.4103/IJO.IJO_2782_22 PMC10276716

[6] O’Neil, E. C., Henderson, M., Massaro-Giordano, M. & Bunya, V. Y. Advances in dry eye disease treatment. *Curr. Opin. Ophthalmol.* **30**(3), 166–178 (2019). 30883442 10.1097/ICU.0000000000000569 PMC6986373

[7] Yang, L., Li, J., Zhou, B. & Wang, Y. An injectable copolymer for in situ lubrication effectively relieves dry eye disease. *ACS Mater. Lett.* **7**, 884–890 (2025).

[8] Ridder, W. H. III. & Karsolia, A. New drugs for the treatment of dry eye disease. *Clin. Optomet.* **153**, 91–102 (2015).

[9] Mondal, H., Kim, H. J., Mohanto, N. & Jee, J. P. A review on dry eye disease treatment recent progress, diagnostics, and future perspectives. *Pharmaceutics* **15**(3), 990 (2023). 36986851 10.3390/pharmaceutics15030990 PMC10051136

[10] Lemp, M. A. Advances in understanding and managing dry eye disease. *Am. J. Ophthalmol.* **146**(3), 350–356 (2008). 18599017 10.1016/j.ajo.2008.05.016

[11] Ling, J. et al. Current advances in mechanisms and treatment of dry eye disease: toward anti-inflammatory and immunomodulatory therapy and traditional Chinese medicine. *Front. Med.* **8**, 815075 (2022). 10.3389/fmed.2021.815075 PMC8801439 35111787

[12] Hantera, M. M. Trends in dry eye disease management worldwide. *Clin. Ophthalmol.* **12**, 165–173 (2021). 10.2147/OPTH.S281666 PMC7814230 33488065

[13] Boudry, C., Baudouin, C. & Mouriaux, F. International publication trends in dry eye disease research: a bibliometric analysis. *Ocular Surf.* **16**(1), 173–179 (2018). 10.1016/j.jtos.2017.10.002 29031646

[14] Seen, S. & Tong, L. Dry eye disease and oxidative stress. *Acta Ophthalmol.* **96**(4), e412–e420 (2018). 28834388 10.1111/aos.13526

[15] Marshall, L. L. & Roach, J. M. Treatment of dry eye disease. *Consult. Pharmac.®* **31**(2), 96–106 (2016). 10.4140/TCP.n.2016.96 26842687

[16] Trinajstic, N. *Chem. Graph Theory* (CRC Press, Boca Raton, FL, 1992).

[17] Gozalbes, R., Doucet, J. P. & Derouin, F. Application of topological descriptors in Gsar and drug design: history and new trends. *Curr. Drug Targ. Infect. Disord.* **2**, 93–102 (2002). 10.2174/1568005024605909 12462157

[18] Roy, Kunal. Topological descriptors in drug design and modeling studies. *Mol. Divers.* **8**, 321–323 (2004). 15612635 10.1023/b:modi.0000047519.35591.b7

[19] Gao, W., Wang, W. F. & Farahani, M. R. Topological indices study of molecular structure in anticancer drugs. *J. Chem.*10.1155/2016/3216327 (2016).

[20] Huang, L., Alhulwah, K. H., Hanif, M. F., Siddiqui, M. K. & Ikram, A. S. On QSPR analysis of glaucoma drugs using machine learning with XGBoost and regression models. *Comput. Biol. Med.* **187**, 109731 (2025). 39879884 10.1016/j.compbiomed.2025.109731

[21] Ravi, V., Siddiqui, M. K., Chidambaram, N. & Desikan, K. On topological descriptors and curvilinear regression analysis of antiviral drugs used in COVID-19 treatment. *Polycycl. Aromat. Compd.* **42**(10), 6932–6945 (2022).

[22] Zhang, X., Reddy, H. G., Usha, A., Shanmukha, M. C., Reza Farahani, M., & Alaeiyan, M. A study on anti-malaria drugs using degree-based topological indices through QSPR analysis, (2022). 10.3934/mbe.2023167 36899594

[23] Arockiaraj, M., Greeni, A. B., & Kalaam, A. A. Comparative analysis of reverse degree and entropy topological indices for drug molecules in blood cancer treatment through QSPR regression models. *Polycyclic Aromatic Compounds*, 1-18 (2023).

[24] Ezekiel, M., & Fox, K. A. Methods of correlation and regression analysis: Linear and curvilinear, (1959).

[25] Sabljić, A. Topological indices and environmental chemistry. *Practical applications of quantitative structure-activity relationships (QSAR) in environmental chemistry and toxicology*, 61-82, (1990).

[26] Hayat, S., Alanazi, S. J. & Liu, J. B. Two novel temperature-based topological indices with strong potential to predict physicochemical properties of polycyclic aromatic hydrocarbons with applications to silicon carbide nanotubes. *Phys. Scripta* **99**(5), 055027 (2024).

[27] Kirana, B., Shanmukha, M. C., & Usha, A. A QSPR analysis and curvilinear regression models for various degree-based topological indices: Quinolone antibiotics. Heliyon (2024). 10.1016/j.heliyon.2024.e32397 PMC11226772 38975153

[28] Havare, Ozge Colakoglu. Topological indices and QSPR modeling of some novel drugs used in the cancer treatment, *Int. J. Quant. Chem.*, 2021, e26813, 1-23.

[29] Shanmukha, M. C., Basavarajappa, N. S., Shilpa, K. C. & Usha, A. Degree-based topological indices on anticancer drugs with QSPR analysis. *Heliyon* **6**, e04235 (2020). 32613116 10.1016/j.heliyon.2020.e04235 PMC7322043

[30] Odu, G. O. Weighting methods for multi-criteria decision making technique. *J. Appl. Sci. Environ. Manag.* **23**(8), 1449–1457 (2019).

[31] Zhang, X., Aslam, A., Saeed, S., Razzaque, A. & Kanwal, S. Investigation for metallic crystals through chemical invariants, QSPR and fuzzy-TOPSIS. *J. Biomol. Struct. Dyn.* **42**(5), 2316–2327 (2024). 37154534 10.1080/07391102.2023.2209656

[32] Tamilarasi, C. & Raj, F. S. Multilinear-regression models developed by four novel degree-based topological Indices in Qspr analysis. *J. Algeb. Statist.* **13**(2), 3173–3181 (2022).

[33] Suresh, M., Tolasa, F. T. & Bonyah, E. QSPR/QSAR study of antiviral drugs modeled as multigraphs by using TI’s and MLR method to treat COVID-19 disease. *Sci. Rep.* **14**(1), 1–14 (2024). 38849399 10.1038/s41598-024-63007-w PMC11711684

[34] Rasheed, M. W., Mahboob, A. & Hanif, I. On QSAR modeling with novel degree-based indices and thermodynamics properties of eye infection therapeutics. *Front. Chem.* **12**, 1383206 (2024). 38860235 10.3389/fchem.2024.1383206 PMC11163131

[35] Zhou, H. et al. On QSPR analysis of molecular descriptor and thermodynamic features of narcotic drugs. *Polycycl. Arom. Compd.* **44**(5), 3079–3099 (2024).

[36] Hui, Z. H., Aslam, A., Kanwal, S., Saeed, S. & Sarwar, K. Implementing QSPR modeling via multiple linear regression analysis to operations research: A study toward nanotubes. *Europ. Phys. J. Plus* **138**(3), 200 (2023). 10.1140/epjp/s13360-023-03817-5 PMC9983549 36883184

[37] Ashraf, T. & Idrees, N. Topological indices based VIKOR assisted multi-criteria decision technique for lung disorders. *Front. Chem.* **12**, 1407911 (2024). 39380949 10.3389/fchem.2024.1407911 PMC11459094

[38] Pandi, U. P., Hayat, S., Marimuthu, S. & Konsalraj, J. Structure-property modeling of pharmacokinetic characteristics of anticancer drugs via topological indices, multigraph modeling and multi-criteria decision making. *Int. J. Quant. Chem.* **124**(11), e27428 (2024).

[39] Li, Y., Aslam, A., Saeed, S., Zhang, G. & Kanwal, S. Targeting highly resisted anticancer drugs through topological descriptors using VIKOR multi-criteria decision analysis. *Europ. Phys. J. Plus* **137**(11), 1245 (2022). 10.1140/epjp/s13360-022-03469-x PMC9667010 36405039

[40] Gutman, I. & Trinajstić, N. Graph theory and molecular orbitals. Total -electron energy of alternant hydrocarbons. *Chem. Phys. Lett.* **17**(4), 535–538 (1972).

[41] Fajtlowicz, S. On Conjectures of Grafitti II. *Congr. Numer.* **60**, 189–197 (1987).

[42] Gutman, I. On hyper-Zagreb index and coindex. Bulletin (Académie serbe des sciences et des arts. Classe des sciences mathématiques et naturelles. *Sci. Math.* **42**, 1–8 (2017).

[43] Furtula, B. & Gutman, I. A forgotton topological index. *J. Math. Chem.* **53**, 213–220 (2015).

[44] Estrada, E., Torres, L., Rodriguez, L. & Gutman, I. An atom-bond connectivity index: Modeling the enthalpy of formation of alkanes. *Indian J. Chem.* **37**, 849–855 (1998).

[45] Randic, M. On Characterization of molecular branching. *J. Am. Chem. Soc.* **97**, 6609–6615 (1975).

[46] Zhou, B. & Trinajstić, N. On a novel connectivity index. *J. Math. Chem.* **46**, 1252–1270 (2009).

[47] Vukičević, D. & Furtula, B. Topological index based on the ratios of geometrical and arithmetical means of end-vertex degrees of edges. *J. Math. Chem.* **46**, 1369–1376. 10.1007/s10910-009-9520-x (2009).

[48] Gutman, I. Geometric approach to degree-based topological indices: Sombor indices. *MATCH Commun. Math. Comput. Chem.* **86**, 11–16 (2021).

[49] Kulli, V. R. Nirmala index. *Int. J. Math. Trends Technol. IJMTT* **67**, 1045 (2021).

[50] Harary, F. *Graph Theory* (Addison-Wesely, Reading Mass, 1969).

[51] Kulli, V. R. *College Graph Theory* (Vishwa Int. Publ, Gulbarga, India, 2012).

[52] Burch, K. J. Chemical applications of graph theory. In *Mathematical Physics in Theoretical Chemistry* (pp. 261-294). Elsevier. (2019).

[53] Wagner, S., & Wang, H. Introduction to chemical graph theory. *Chapman and Hall/CRC*. (2018).

[54] Rasheed, M. W., Mahboob, A. & Hanif, I. On QSAR modeling with novel degree-based indices and thermodynamics properties of eye infection therapeutics. *Front. Chem.* **12**, 1383206 (2024). 38860235 10.3389/fchem.2024.1383206 PMC11163131

[55] Idrees, N., Noor, E., Rashid, S. & Agama, F. T. Role of topological indices in predictive modeling and ranking of drugs treating eye disorders. *Sci. Rep.* **15**(1), 1271 (2025). 39779776 10.1038/s41598-024-81482-z PMC11711676

